# A Treatise on the Diseases of the Chest, Being a Course of Lectures Delivered at the New York Hospital

**Published:** 1852-03

**Authors:** 


*A Treatise on the Diseases of the Chest, being a Course of Lectures delivered at the New York Hospital.* By John A. Swett, M. D., Physician to the New York Hospital.

In our next number, we shall notice at length this work, which has just been issued from the press of Messrs. D. Appleton & Co. of New York.

